# Sexual Segregation and Flexible Mating Patterns in Temperate Bats

**DOI:** 10.1371/journal.pone.0054194

**Published:** 2013-01-24

**Authors:** Ruth L. Angell, Roger K. Butlin, John D. Altringham

**Affiliations:** 1 Faculty of Biological Sciences, University of Leeds, Leeds, West Yorkshire, United Kingdom; 2 Dept. Animal and Plant Sciences, University of Sheffield, Sheffield, South Yorkshire, United Kingdom; University of Western Ontario, Canada

## Abstract

Social structure evolves from a trade-off between the costs and benefits of group-living, which are in turn dependent upon the distribution of key resources such as food and shelter. Males and females, or juveniles and adults, may have different priorities when selecting habitat due to differences in physiological or behavioural imperatives, leading to complex patterns in group composition. We studied social structure and mating behaviour in the insectivorous bat *Myotis daubentonii* along an altitudinal gradient, combining field studies with molecular genetics. With increasing altitude the proportion of males in summer roosts increased and only males were present in the highest roosts. With increasing altitude environmental temperature decreased, nightly variation in temperature increased, and bat foraging activity decreased, supporting the hypothesis that the harsher, high elevation sites cannot support breeding females. We found that offspring in female-dominated lowland roosts had a very high probability of being fathered by bats caught during autumn swarming at hibernation sites, in contrast to those in intermediate roosts, which had a high probability of being fathered by males sharing the nursery roost with the females. Whilst females normally appear to exclude males from nursery colonies, for those in marginal habitats, one explanation for the presence of males is that the thermoregulatory benefits to the females may outweigh disadvantages, such as competition for food, and give some males an opportunity to increase their breeding success. We suggest that the environment, and its effects on resource distribution, thus determine social structure, which in turn determines the mating pattern that has evolved.

## Introduction

Social structure evolves from a trade-off between the costs and benefits of group-living, which are in turn dependent upon the distribution of key resources such as food and shelter. Males and females, or juveniles and adults, may have different priorities when selecting habitat due to differences in physiological or behavioural imperatives, leading to complex patterns in group composition [1] [2] [3]. Senior *et al.* [4] showed that sexual segregation in the bat *Myotis daubentonii* along an altitudinal gradient also led to segregation among groups of males. At high elevations only males were present in habitat unable to support the high energetic demands of nursing females. At mid elevations males shared nursery roosts with females and had a much greater chance of fathering offspring from these roosts than the males at higher elevations. These males were presumed, on the balance of evidence, to exclude other males from habitat and roosts occupied by females. The excluded males were, however, able to mate during autumn swarming, but with a much lower probability of fathering the young from mid elevation roosts. Swarming occurs during the typically brief visits bats make to hibernation sites in late summer and autumn to mate. As the swarming season progresses into hibernation an increasing proportion of the visiting bats, of both sexes, remain in the hibernation sites e.g. [5] [6] where it is possible that mating continues through the winter. Here, we address three questions raised by Senior *et al.* [4]: (1) Other studies suggest that mating during swarming, not summer in roosts, is the primary sexual behaviour in *Myotis* species. Can these apparently conflicting results be reconciled? (2) *Myotis daubentonii* nursery colonies in the lowlands comprise almost exclusively adult females and their young. In the absence of a dominant male group in the roost, what is the mating behaviour, as assessed by patterns of paternity? (3) Can this variation in roosting and mating behaviour be explained on the basis of habitat and resources?

Daubenton's bat, *Myotis daubentonii*, is a small insectivorous species that feeds over smooth water, catching insects from the air or the water surface. In summer it roosts in trees, buildings and bridges close to water. In late summer and autumn, prior to hibernation, *Myotis* species swarm at caves and other underground sites e.g. [7], [6], [8] and swarming is believed to be the primary mating behaviour of most *Myotis* species e.g. [9] [10] [5] [11]. We studied the same ringed population in the Yorkshire Dales National Park, UK, investigated by Senior *et al.* [4]. Full details can be found in the earlier paper. The absence of females at high elevations, a widely observed phenomenon in temperate bats e.g. [12] [13], can be explained by the high energetic demands of reproduction which cannot be met by sub-optimal foraging conditions [12]. Males have lower energy requirements and the ability to use facultative heterothermy (torpor) to make substantial energy savings e.g. [14], an option not open to breeding females since it reduces foetal growth rates and possibly milk production [15]. To address the three questions posed above, we extended the study downstream of the sites studied by Senior *et al.* [4], investigating the changing patterns of roost composition, social structure and paternity, in relation to environment. Lowland nurseries typically have few resident males, suggesting that the higher proportion of mating in summer roosts compared to swarming observed by Senior *et al.* [4] may be a feature only of populations at mid elevations. Our hypothesis was that a flexible mating pattern has evolved to fit the prevailing social structure, itself a result of varying environmental conditions. We predicted that swarming would be the dominant sexual behaviour of lowland populations.

## Materials and Methods

### Ethics statement

All bats are protected under UK and EU law. Bats were caught and ringed (banded) under licence from Natural England, the statutory nature conservation organisation and wing biopsies taken under licence from both Natural England and the UK Home Office. Full methods are given below.

### Study site

**Figure 1:**
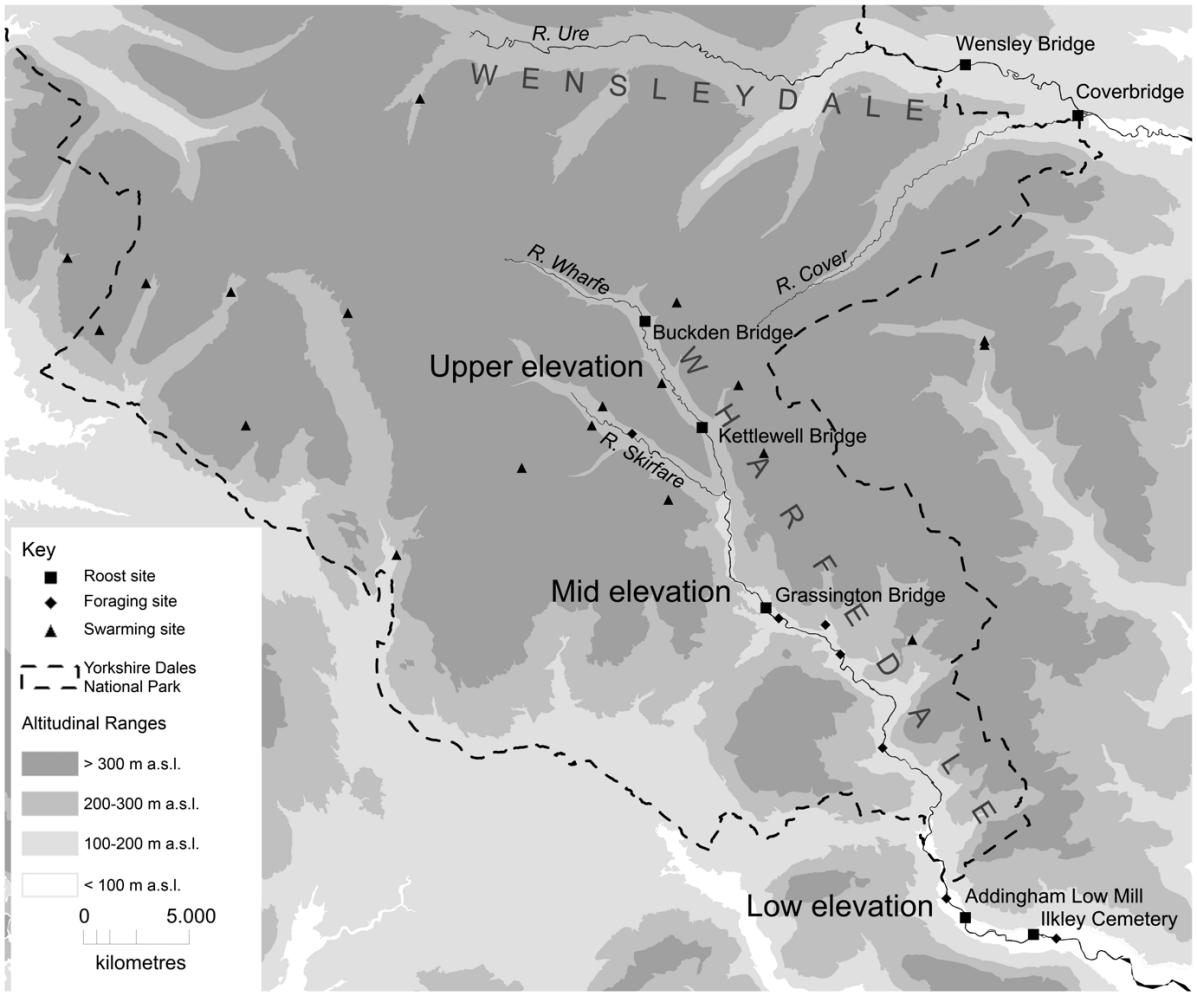
Map of the Yorkshire Dales National Park, UK. Study area with locations of summer roost, foraging and swarming sites.

The study area was a 40 km stretch of the River Wharfe in the Yorkshire Dales National Park, UK (latitude 54°N) (Figure 1). The river falls from 260 m to 70 m a.s.l. along the study site. The upper-elevation site, (>200 m a.s.l.) is a narrow post-glaciation valley with steep sides. The river is <5 m wide, frequently shallow and turbulent, with rocks breaking the surface. The mid-elevation site (100–200 m a.s.l.) is wider and the river is broader, deeper and smoother. At the low-elevation site (<100 m a.s.l.) the river is typically 20 m wide and smooth. Land-use bordering the river is pasture with some broadleaved woodland.

### Acoustic surveys and environmental monitoring

Temperature loggers (TinyTag TGP-4500, www.geminidataloggers.com) were placed in upper-, mid- and low-elevation sites (Kettlewell Bridge, Grassington Bridge and Addingham Low Mill, see Figure 1) 1 m from the ground and 2 m from the river, sheltered from sun and rain. Temperature was recorded every 30 minutes from 6 pm to 6 am, June–August 2005.

Acoustic surveys were conducted on 18×1 km walked transects along riverside footpaths. Each transect was walked (at approximately 3.5 km h^−1^) upstream and downstream on the same night starting one hour after sunset. Time-expanded recordings were made from Pettersson D240x bat detectors to Edirol R-09 digital recorders. Detectors were directed to pick up calls from bats flying over the water surface, maximising the chance of *Myotis* calls being from *M. daubentonii*. Transects were carried out over two weeks in July 2007 on warm, dry evenings (>8°C) with little or no wind. Sonograms were viewed using BatSound (www.BatSound.com). All *Myotis* calls were assumed to be *M. daubentonii*. Of 272 *Myotis* bats caught over rivers in the area between 1996 and 2006, 87% were *M. daubentonii*. Results were expressed as bat passes or feeding buzzes km^−1^.

### Bat capture

Bats were caught and ringed (banded) under licence from Natural England, the statutory nature conservation organisation and wing biopsies taken under licence from both Natural England and the UK Home Office. Bats were captured at summer roosts with static hand nets and at swarming and foraging sites using harp traps and mist nets. Genetic data are from bats captured between July 2004 and August 2007, other data were collected between 1996 and 2007. Mist nets were monitored continuously and bats removed immediately on entry. Harp traps were inspected at least every 15 min and all bats removed at each inspection. Bats were hung in a safe place in cotton bags prior to processing. All bats were processed and released at the site of capture within 1 h. Bats were weighed, forearm length was measured, and a numbered aluminium ring (supplied by the Mammal Society, UK) was placed on the right forearm of each bat. A 3 mm biopsy was taken from each outstretched wing using a sterile biopsy punch over a sterilised plastic board. Biopsies were stored in 100% ethanol prior to analysis. Age was classed as either juvenile (born that year) or adult (born the previous year or earlier) [16]. The ‘chin-spot’ [17] was not used to distinguish between adults and juveniles as some individuals retained it for up to at least four years.

### Genotyping

Data were from individuals caught between 2004 and 2006 and independent of those used by Senior *et al.* [4]. Genomic DNA extraction and PCR methods are given in Methods S1. Ten polymorphic microsatellite loci were used in DNA amplification and samples were genotyped using GeneMapper 3.7 (Applied Biosystems). Locus and allele information is provided in Methods S1.

### Paternity assignment

Two methods of paternity assignment were used. The first method determined the likelihood of paternity of individual males, based on comparisons of genotypes of offspring and putative parents [18] (see Methods S1). This works well for closed populations where mothers are known, candidate numbers of fathers are known and it is possible to genotype a high proportion of candidate fathers. However, in this study mothers were not known and there was a large and unknown number of potential fathers resulting in only a small number of confident parentage assignments. The second method used a Bayesian approach to assign probabilities of parentage to male groups, rather than individuals. This allows more effective use of all data available to test hypotheses concerning the prevalent mating pattern.

#### (a) Paternity assignment to individual males

Direct paternity assignment of lower dale and lowland offspring to individual males was carried out using Cervus 3.0 [18]. Data for each year and area were analysed twice: *Full analyses* with males from Wharfedale and Wensleydale summer sites and Yorkshire Dales swarming sites, to determine where the paternity assignments were most likely to lie; and *Wharfedale analyses* with males from Wharfedale summer sites only to reduce candidate number and increase likelihood ratio, gaining a clearer picture of where fathers were most likely to be within Wharfedale. Full details are provided in Methods S1.

#### (b) Paternity assignment to male group

Burland *et al.* [19] used a Bayesian approach to assign probabilities of parentage to groups, rather than individuals. Senior *et al.* [4] modified their program to estimate the mating success of males from four groups in relation to offspring born at a single mid-elevation nursery colony. We adapted the program further to estimate the mating success of males from four groups in relation to offspring born in the low-elevation roosts. Observed genotypes were used to calculate the probability that a low-elevation offspring was the product of a mating between any one of the sampled or unsampled females from a low-elevation roost, and any one of the sampled or unsampled males from any of four groups (upper-, mid- and low-elevation roosts and swarming). The main modifications were the redefinition of male groups, incorporating the effect of sampling offspring in three years and adjusting mutation rates. Full details are in Methods S1. In brief, the likelihood of the data (the probability of seeing the observed genotypes, given the model) is the product of the likelihood of observing the offspring genotypes given the genotypes of potential parents (the probability of offspring genotypes, given the parental groups), and the likelihood of each possible pairwise combination of male and female parents from specified groups (the probability of parents, given the model). The latter is a function of a set of model parameters (θ) consisting of the numbers of males and females in each group (typed and untyped individuals) and the probabilities of the father and mother being from the groups in question. The posterior distributions of θ were estimated using the Metropolis algorithm, a Markov chain Monte Carlo method. The prior distributions of the parameters (θ) are specified in Methods S1. All ten microsatellite loci were used in the analysis and the program took account of the sex-linkage, error rates and mutation rates specific to loci. The program was run for 100,000 iterations, including a 10,000 sample burn-in period. Additional runs were made from different starting points and with relaxed prior distributions, to ensure the results were robust, and a null model was run with equal probability of paternity per male, regardless of group. Full details are in the Methods S1.

## Results

### Temperature change down the dale

Mean summer night time temperature increased with decreasing altitude (one-way ANOVA; F_(2,183)_ = 9.017; P<0.001), with the low-elevation site being on average 1°C warmer than the mid- (Tukey test; P = 0.006) and upper- (Tukey test; P<0.001) elevation sites. The upper-elevation site experienced significantly greater average nightly temperature variation (4°C) than the mid- (2°C) (Kruskal-Wallis test; Z = −5.91; P<0.001) and low- (2.3°C) elevation sites (Kruskal-Wallis test; Z = −4.75; P<0.001).

### Bat activity

The number of bat passes was highly correlated with the number of feeding buzzes when all transects were pooled (Spearman's Rank test; n = 18; r = 0.834; P<0.001), justifying the use of bat passes as a measure of foraging activity (pass∶buzz ratio approx. 10). Bat activity declined significantly with increasing altitude (df = 16; R^2^ = 0.2378; P = 0.04, see Figure S1).

### Bat morphology

Pairwise comparisons with Bonferroni adjustment for multiple comparisons following ANCOVA (with area and month as fixed factors and forearm length as a covariate; F_(2,106)_ = 6.094; P = 0.003) showed upper-elevation males were significantly lighter than mid- (P = 0.001) and low- (P = 0.035) elevation males.

### Roost composition down the dale

**Figure 2:**
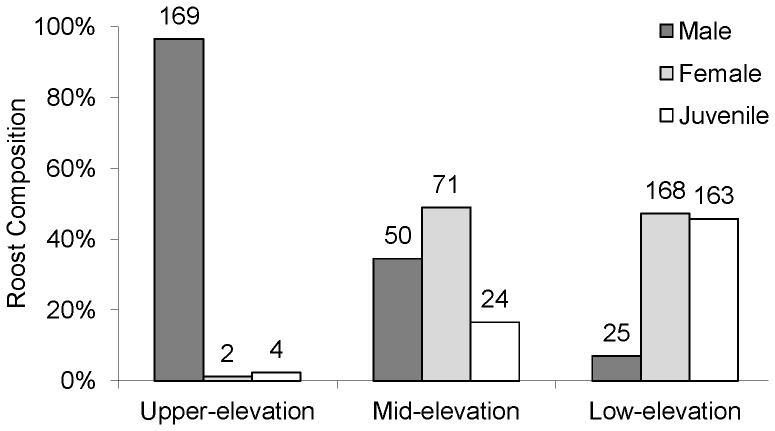
Change in roost composition along the River Wharfe. Data from June–August 1996–2007 (upper-elevation n = 175, mid-elevation n = 145, low-elevation n = 356). Numbers are for unique (ringed) bats caught over the period.

Roosts were in tree holes close to the riverbank, in gaps in the stonework of the many old bridges that cross the river, and in old stone buildings close to the river. Colonies used more than one roost. Roost composition (data from bats caught 1996–2007) changed markedly down the valley (Figure 2). Upper-elevation roosts were almost exclusively composed of males. Mid-elevation roosts had a sex ratio closer to unity with a small proportion of juveniles. Low-elevation roosts consisted almost equally of females and juveniles with few males. Significantly more males than females were caught at roosts (m∶f = 169∶2, *χ^2^* = 161, P<0.001) in the upper-elevation sites. At mid-elevation differences at roost sites (50∶71, *χ^2^* = 3.31, ns) were not significant. Significantly more females were caught at roosts (25∶168, *χ^2^* = 104, P<0.001) at low elevations. All Chi-squared tests were with Yates' correction for continuity (df = 1). Qualitatively similar patterns were observed in foraging bats, but too few were captured for meaningful analysis.

### Philopatry

Approximately half of adults (both males and females) ringed at a particular roost were recaptured there in subsequent years. Only 4% of adult males and <2% of adult females were recaptured at a different roost. Only 2% of adult males and no adult females were ever recaptured outside the area (i.e. upper-, mid or low-elevation sites) in which they were ringed. Half (50%) of ringed juvenile males that were recaptured had left their natal area, but no juvenile females were observed to have moved (full details in Table S1).

### Paternity assignment to individual males

Sample sizes of genotyped individuals were as follows. 138 offspring: 10 from the mid-elevation roost for comparison with Senior *et al.* [4] and 128 from the low-elevation site. 163 females: 14 from mid-elevation and 149 from the low-elevation roosts. The ‘full’ analyses used 341 males from all roosts and swarming sites. The ‘Wharfedale’ analyses used 133 males from the intensively studied valley of the River Wharfe alone. Full details are given in Methods S1.

Of 10 mid-elevation offspring, two could be assigned fathers (one with strict confidence (95%), one relaxed (80%)). Both fathers had previously been caught at roosts outside the area (one in an upper-elevation roost and low-elevation roosts, the other at a roost in an adjacent valley, Wensleydale (Figure 1)). For the 128 low-elevation offspring, 10 fathers were assigned with strict confidence, six with relaxed confidence and 12 as part of parent pairs with relaxed confidence. These males had been caught at upper-, mid- and low-elevation roosts and swarming sites. Full details are provided in Methods S1.

### Paternity assignment to male group

**Figure 3:**
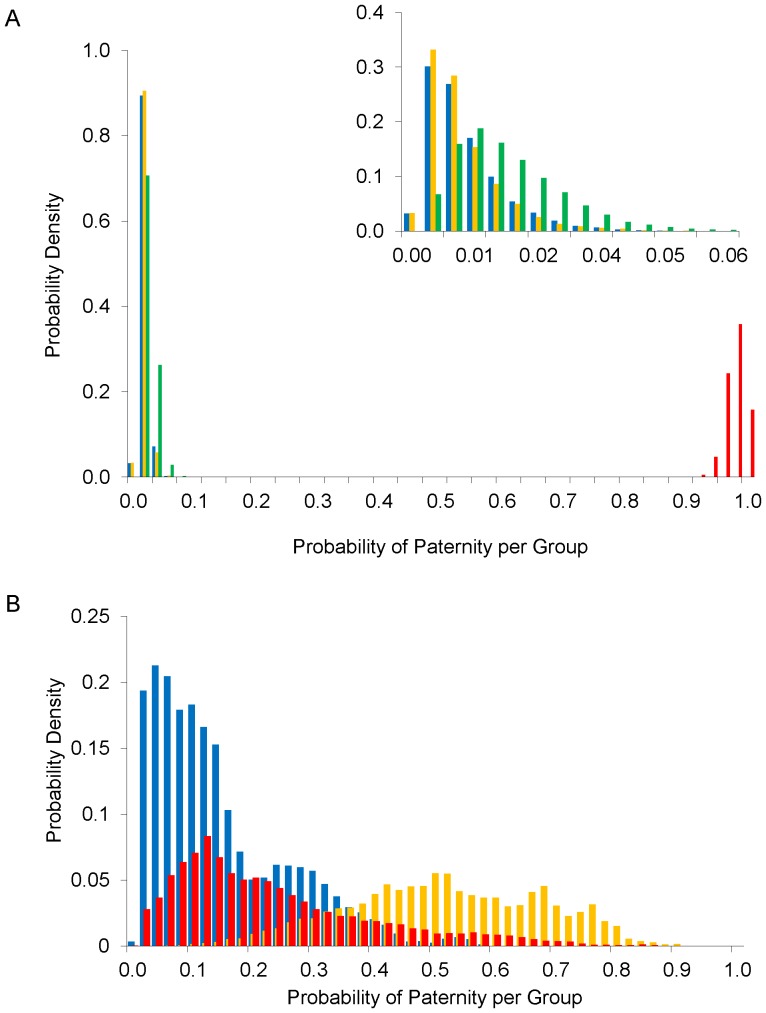
Posterior distributions for paternity probabilities at the group level. Posterior distributions for the probabilities that fathers (at the group level) came from roosts in the (blue) upper-elevation, (yellow) mid-elevation and (green) low-elevation, and from (red) swarming sites. For (A) low-elevation offspring (the inset graph shows the Wharfedale roost posterior distributions in greater detail), and (B) mid-elevation offspring (adapted from [4]).

Sample sizes and population sizes used in the analysis are given in Methods S1. In summary the analysis included genotypes of 307 males (209 swarming, 63 upper-, 19 mid- and 16 low-elevation), together with data from 149 females and 128 offspring from low-elevation sites. Group assignment results indicated that swarming males were responsible for fathering most of the low-elevation offspring (Figure 3a). The probabilities that fathers of low-elevation offspring were from Wharfedale summer roosts were all <5%, the probabilities that fathers were from the swarming group were all >95%. In contrast, Figure 3b (adapted from [4]) shows that fathers of mid-elevation offspring were more likely to be from the mid-elevation roost, not from swarming sites.

**Figure 4:**
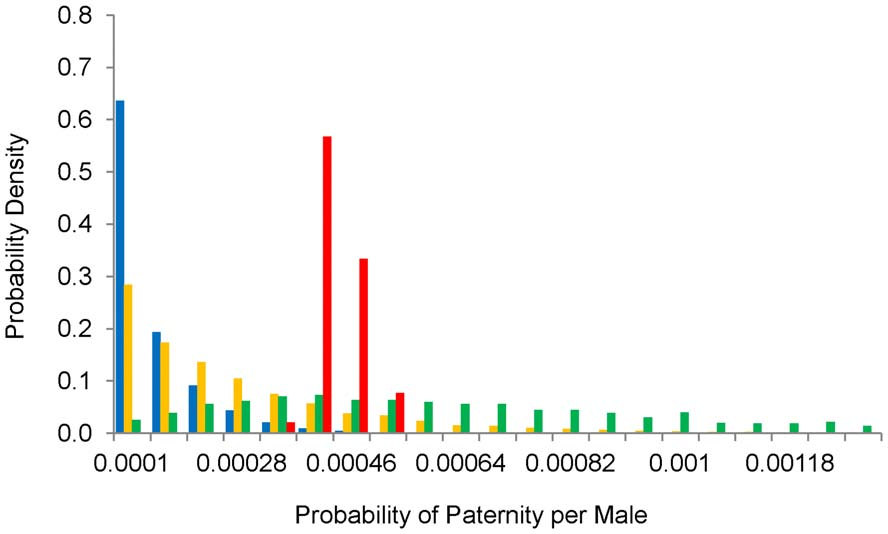
Posterior distributions for paternity probabilities at the individual level. Posterior distributions for the probabilities that individual males from the (blue) upper-elevation, (yellow) mid-elevation and (green) low-elevation roosts and (red) swarming sites fathered low-elevation offspring.

Swarming males (model estimate N = 2,500) greatly outnumbered those in the upper- (90), mid- (31) and low-elevation (25) summer roosts. Although the analysis inferred that most fathers of low-elevation offspring were swarming males, individual low-elevation roost males may actually have had the highest chance of fathering one of these offspring because there were so few of them. This is illustrated in Figure 4, which shows the probabilities of paternity per male from each group. Swarming and roost groups are not mutually exclusive: the swarming group includes males from our focal roosts and many males from other roosts throughout Wharfedale and neighbouring valleys. However, the mating probabilities reported for swarming males exclude the contribution from males in our sampled roosts.

The results were robust to starting conditions and independent of the width of the prior distributions. The definitive model was favoured over the null model, with invariant probability of paternity per male, (Bayes factor = 3.1 based on the harmonic means of post burn-in log likelihoods, see Results S1 for further details). Individual assignment patterns (Results S1) did not contradict the group assignment results, but individual assignments were too few to deduce relative paternity patterns.

## Discussion

We show that offspring from low-elevation nursery roosts are fathered primarily during autumn swarming. Using the same approach Senior *et al.* [4] showed that offspring from the mid-elevation nursery roost (the highest elevation nursery) were primarily fathered by resident males. This difference suggests that mating strategy adapts to fit social structure and this in turn has evolved in response to environmental differences.

At low-elevation sites, temperatures are at their highest and most stable and will support large and stable insect populations, hence the higher foraging activity we observed. The increasing width and smoother surface of the river at low elevations will also improve foraging conditions. Nursery colonies are large since the home range can support large numbers of bats. Abundant food and the thermoregulatory benefits provided by large numbers of bats in the roosts reduce the need for torpor and facilitate homeothermy, increasing reproductive success. Males may be largely excluded from roosts and even foraging sites, since they compete for food [20]. A few males may be tolerated in the roost, which increases their opportunity to father offspring, as demonstrated by the individual paternity estimates and the probabilities of paternity per male. However, the paternity assignment to male groups shows that most of the successful mating involves males caught at swarming sites. This strongly suggests that swarming is the primary mating behaviour, as it is for other temperate *Myotis* species (see below).

In mid-elevation roosts, climatic and habitat conditions are less favourable (see temperature and bat activity in Results, and [21] for habitat changes) and food supply is probably more variable. This is reflected in the smaller size of nursery colonies: the home range of a nursery colony can support only a limited number of females. These roosts have a higher proportion of males than the low-elevation roosts. One explanation is that males may be tolerated for the thermoregulatory benefits they bring to smaller colonies in cool roosts in stone bridges and tree holes. Because these roosts have a large proportion of males, resident males are able to father a large proportion of the offspring. These males, in common with all other males, also have the opportunity to mate at swarming sites later in the season, and this was confirmed by ringing.

At upper-elevations only males are found, because the environment is not able to support the energetic demands of reproductive females e.g. [12]. These males are either excluded from lower elevations or are avoiding more intense competition for resources downstream. After correcting for skeletal size, these males are lighter (this study and [4]) and must forage for longer periods and over greater distances than males at lower elevations [4], suggesting that they are excluded from more favourable foraging sites downstream. These males are able to mate at swarming sites and it is the swarming population, comprising bats from summer roosts in the valley and beyond, that fathers most of the offspring from the large low-elevation populations where nursery colonies are predominantly female.

In summary, we found that most offspring are fathered during autumn swarming. However, the breeding success of a small proportion of males is improved because they live with females in nursery roosts during late summer. Whilst females normally appear to exclude males from nursery colonies, for those in marginal habitats, the thermoregulatory benefits may outweigh disadvantages, such as competition for food.

Swarming is a widespread mating mechanism that facilitates gene flow and helps maintain genetic diversity among temperate bats e.g. [22] [10] [23]. Other studies support the view that mating occurs outside summer habitat, consistent with mating at swarming sites e.g. [24] [25] [19]. However, it is clear that in other species too, more than one mating strategy may be in operation. For example, *Myotis bechsteinii* shows similarities to lowland *M. daubentonii*: male *M. bechsteinii* offspring disperse from their natal colonies and half the males roosting in close proximity to nursery colonies are immigrants [26] [27] [28], but these local males father <25% of the offspring born at these colonies, implying that the rest are fathered at swarming sites [22] [29]. Although such studies show inter-specific variation in mating pattern, we believe this study is the first to explain geographical differences on the basis of environmental factors. Habitat fragmentation and climate change, in changing prey distribution and roost microclimate, are likely to affect these complex, large-scale behavioural patterns. Is behaviour sufficiently flexible to deal with such change?

## Supporting Information

Methods S1 **Methods including genomic DNA extraction and PCR methods, primer and PCR reaction details and properties for each locus, and paternity assignment to individual males and to male group.**
(DOCX)

Results S1 **Results for paternity assignment to individual males and male groups.**
(DOCX)

Figure S1 **Relationship between bat activity and altitude.** The relationship between bat activity and altitude based on 18×1 km walked acoustic transects along riverside footpaths in July 2007. Bat activity declined significantly with increasing altitude (df = 16; R^2^ = 0.2378; P = 0.04). Full details are given in the main article.
(DOCX)

Table S1 **Summary of ringed and recaptured bats.** Numbers (*N*) of *M. daubentonii* (A) adult males and (B) adult females that were ringed at named Wharfedale roost sites, with the number (*N*) and proportion (%) which were recaptured at the same roost, or at a different roost.
(DOCX)
