# Supplementary material for: Sexual Segregation and Flexible Mating Patterns in Temperate Bats
Source: PLoS One. 2013 Jan 24;8(1):e54194. doi: 10.1371/journal.pone.0054194 (PMC3554751; doi:10.1371/journal.pone.0054194)
Supplement: Methods S1 — Methods including genomic DNA extraction and PCR methods, primer and PCR reaction details and properties for each locus, and paternity assignment to individual males and to male group. (DOCX) [file pone.0054194.s001.docx]

**Supporting Information Methods S1**

**Sexual segregation and flexible mating patterns in temperate bats**

**RA Angell, RK Butlin and JD Altringham**

A small amount of information has been repeated from the main text to ensure that the information is comprehensible without cross reference to the paper.

**METHODS**

**Genomic DNA extraction and PCR methods**

Genomic DNA was extracted from wing membrane using a silica plate extraction method (Whitlock *et al.*, 2008). The DNA yield was measured by fluorimetry (Fluostar Optima, BMG Labtechnologies) and sample diluted to 1 ng/μl for the PCR reaction. Ten microsatellite primer pairs developed for use in other bat species were used in the amplification of DNA. All loci are polymorphic and suitable for cross-species amplification in *M. daubentonii* (Senior *et al.*, 2005). One primer of each pair was fluorescently labelled at the 5’ end (Applied Biosystems, Web Scientific, Crewe, UK). The other primer had a 5’ tail addition (Applied Biosystems) to reduce errors in sizing the PCR product (Brownstein *et al.*, 1996). PCR reactions were carried out on a Tetrad thermal cycler. The 10μl reactions comprised 1μl of 1 ng/μl DNA, 1 μl of 10x *Taq* buffer, 0.3 μl of 1.5 mM MgCl_2_, 1 μl of 0.2 mM dNTPs, 0.05 or 0.025 μl of *Taq* polymerase, and 1 μl of 1 μM forward and reverse primer. 1 μl of 10 % DMSO (to reduce stutter bands) and 1 μl 0.5 μg/μl BSA (to inhibit contaminants) were added. PCR conditions were: 95 °C for 3 minutes; 35 cycles of 94 °C for 30 seconds, primer-specific annealing temperatures for 30 seconds and 72 °C for 30 seconds; then 72 °C for 10 minutes, 10 °C for 10 seconds. The conditions for the Touchdown PCR reactions (to reduce spurious priming and stutter bands, Don *et al*. 1991) were: 95 °C for 3 minutes, 10 cycles of 94 °C for 30 seconds, primer-specific annealing temperatures minus 1 °C per cycle for 30 seconds, 72 °C for 30 seconds; then 35 cycles of 94 °C for 30 seconds, primer-specific annealing temperatures for 30 seconds, 72 °C for 30 seconds; then 72 °C for 10 minutes, 10 °C for 10 seconds. PCR conditions were optimized for each locus (Table 1). The PCR product was diluted and run with a size marker (ROX) on an ABI3730 48-well capillary sequencer (Applied Biosystems). Allele ranges and fluorescent dyes allowed for a post-PCR multiplex of two groups of five loci. On each plate there were five control samples to increase awareness of contamination or inconsistency in allele sizes between gels: two samples repeated on the same plate, two samples of known genotype which were run on every plate, and one negative control (no DNA). Samples were genotyped using GeneMapper version 3.7 (Applied Biosystems), see Table 2 for allele information. Source references can be found in Senior *et al*. (2005).

**Table 1. Primer and PCR reaction details for each locus.**

Locus name; repeat motif of microsatellite region; fluorescent label; quantity of TAQ (μl) added to PCR reaction; addition of BSA and/or DMSO to PCR reaction; annealing temperature of PCR program or TD for touchdown program with temperature range; *M.G.* is post-PCR multiplex group; *allele range* is number of base-pairs; Paur03* is sex-linked on X-chromosome.

| **Locus** | **Repeat Motif** | **Fluor. Label** | **TAQ (μl)** | **BSA & DMSO** | **Annealing Temp. (°C)** | **M.G.** | **Allele Range (bp)** |
| --- | --- | --- | --- | --- | --- | --- | --- |
| B15 | (CT)_16_ (CA)_21_ | HEX | 0.05 | - | 45 - 55 (TD) | 1 | 163 - 200 |
| B22 | (GT)_10_ GA (GT)_5_ | 6-FAM | 0.025 | DMSO | 60 | 1 | 158 - 195 |
| C113 | (ACC)_7_ | HEX | 0.025 | DMSO | 50 - 60 (TD) | 1 | 98 - 109 |
| D15 | (AC)_17_ | NED | 0.025 | DMSO | 60 | 1 | 100 - 138 |
| D9 | (CT)_29_ | NED | 0.025 | Both | 56 | 2 | 114 - 164 |
| E24 | (TC)_32_ | NED | 0.05 | Both | 60 | 2 | 212 - 259 |
| G9 | (TC)_19_ | HEX | 0.025 | Both | 55 | 2 | 148 - 218 |
| H19 | (GT)_18_ CT (GT)_2_ | 6-FAM | 0.025 | - | 60 | 2 | 80 - 113 |
| H29 | (CA)_21_ | 6-FAM | 0.025 | - | 46 - 56 (TD) | 2 | 167 - 199 |
| Paur03* | (TG)_17_ | 6-FAM | 0.025 | - | 60 | 1 | 230 - 256 |

**Table 2.** **Properties of loci.**

Locus name; *N* is number of samples; *A* is number of alleles; *H*_o_ is observed heterozygosity; *H*_e_ is expected heterozygosity; *F*_IS_ is inbreeding coefficient; *HWE* is significance of deviation from Hardy-Weinberg equilibrium where *ns* is not significant and ** is highly significant (P < 0.01); and null allele frequency; Paur03* is sex-linked on X-chromosome, and so only females were analysed for this locus.

| **Locus** | **N** | **A** | **H_O_** | **H_E_** | **F_IS_** | **HWE** | **Null allele frequency** |
| --- | --- | --- | --- | --- | --- | --- | --- |
| B15 | 649 | 20 | 0.855 | 0.864 | 0.010 | NS | 0.003 |
| B22 | 653 | 18 | 0.847 | 0.878 | 0.035 | NS | 0.018 |
| C113 | 670 | 7 | 0.461 | 0.465 | 0.008 | NS | 0.003 |
| D15 | 615 | 22 | 0.841 | 0.900 | 0.066 | ** | 0.033 |
| D9 | 656 | 24 | 0.878 | 0.912 | 0.037 | NS | 0.018 |
| E24 | 643 | 28 | 0.871 | 0.909 | 0.042 | NS | 0.021 |
| G9 | 650 | 32 | 0.925 | 0.931 | 0.007 | NS | 0.003 |
| H19 | 654 | 17 | 0.861 | 0.881 | 0.023 | NS | 0.012 |
| H29 | 651 | 15 | 0.896 | 0.891 | -0.005 | NS | -0.003 |
| Paur03* | 240 | 12 | 0.833 | 0.824 | -0.011 | NS | -0.008 |

**Paternity assignment to individual males**

Direct paternity assignment of lower dale and lowland offspring to individual males was carried out using Cervus 3.0 (Kalinowski *et al.*, 2007). The Delta statistic (∆) was used as a criterion for assignment: the difference in LOD scores (natural logarithm of the likelihood ratio over all loci) between the most likely and the second most likely candidate parent. Simulations estimated critical values of ∆ so that the confidence of parentage assignments with real data could be evaluated statistically. Since neither parent was known, parent pair analysis was used to assign the most likely father, mother and parent pair to each offspring. Mother and offspring data were divided into groups according to area (mid-elevation or low-elevation) and year (2004, 2005 or 2006), and analyses were run on each group separately. Offspring later caught as adults were considered as potential parents and re-entered into the analysis. It was not possible to identify mother-offspring pairs by field observations because roosts were inaccessible. It was assumed that mother and offspring shared the same roosts so potential mothers were those caught at roosts within the same area. Males were included regardless of the site at which they were caught. Data for each year and area were analysed twice:

- **Full analyses** with males from Wharfedale and Wensleydale summer sites and Yorkshire Dales swarming sites, to determine where the paternity assignments were most likely to lie.
- **Wharfedale analyses** with males from Wharfedale summer sites only to reduce candidate number and increase likelihood ratio, gaining a clearer picture of where fathers were most likely to be in Wharfedale.

Cervus assignment simulations were run for 100,000 offspring to compile critical ∆ values at 95% (strict) and 80% (relaxed) confidence levels for mid-elevation and low-elevation and years 2004, 2005 and 2006. Parent pair analysis was carried out to assign the most likely father, mother and parent pair to each offspring in each area and year. Simulations and analysis were repeated three times with the same parameters, and repeated with adjusted parameters for 10,000 cycles to test for consistency and robustness.

**Simulation parameters**

The simulation used allele frequencies and the number of candidate parents to be tested for each offspring, taking into account the completeness of genetic typing and the estimated frequency of genotyping error, the number of candidate males and females and the proportion of these typed, and whether there were relatives of the offspring among candidate parents.

Individuals with a minimum of six typed loci were included and the overall proportion of loci typed was greater than 95% across the whole dataset. Genotyping error rate was calculated as the proportion of allelic drop-outs and false alleles and entered into the simulation at 1.1% (the proportion of loci mistyped based on repeat scoring of 10% of the individuals genotyped). This mistyping rate was used as the error rate in the likelihood calculations.

**Candidate numbers**

Candidate numbers represent the pool from which potential mothers and fathers may be drawn, and include both sampled and unsampled individuals. Male and female candidate numbers for ‘Wharfedale analyses’ were derived from the mean population size estimates for Wharfedale roosts. Summer roost population size estimates were calculated using Jolly (Pollock *et al*., 1990) with the Jolly-Seber Model ‘A’ for open populations allowing for death and immigration selected using a goodness-of-fit test. For each roost site, the data for the catch history records were pooled and each capture event was entered either as a year or as a month if there were too few years of catch history data (low-elevation roosts). Population size estimates were 44 for females at the mid-elevation roost, 165 for females at low-elevation roosts, and 146 for males at Wharfedale summer sites. Minimum population size estimates for each group were the products of the number of individuals caught between 2004 and 2006, and the proportion of captured males (or females) in the area. The swarming population size estimate was calculated from the capture rate at swarming sites of males also caught in Wharfedale, i.e. the combined Wharfedale roost mean size estimates were divided by the proportion of males caught at swarming sites that were also caught at Wharfedale roost sites. A total of 21 males were caught at both Wharfedale roosts and swarming sites out of a total of 287 males caught at swarming sites, giving a proportion of 0.073. The combined mean estimates of male population size at Wharfedale roosts was 146, and this value was divided by 0.073 to give an estimate of 1993 for the size of the total male swarming population, including those that roost in Wharfedale in the summer (i.e. the number of candidate males that might have had contact with Wharfedale females and therefore be potential fathers). This was used as the ‘Full analyses’ candidate number of males that could potentially be fathers. The estimate of swarming group males only (1993 minus 146) was 1847. Proportions of candidate parents typed varied for each year and area analysis, depending on the number of samples, and are detailed in Table 3.

**Table 3.** **Paternity assignment to individual males.**

Summary of sample sizes, candidate numbers and proportion of candidate parents typed for each of the twelve simulations run in CERVUS.

**(A) Full analyses**

| **Offspring roost** | **Year** | **Number of samples** | | | **Candidate number** | | **Proportion of candidates sampled** | |
| --- | --- | --- | --- | --- | --- | --- | --- | --- |
|  |  | **Males** | **Females** | **Offspring** | **Males** | **Females** | **Males** | **Females** |
| Mid-elev. | 2004 | 136 | 1 | 2 | 1993 | 44 | 0.068 | 0.023 |
|  | 2005 | 243 | 4 | 2 | 1993 | 44 | 0.122 | 0.091 |
|  | 2006 | 341 | 14 | 6 | 1993 | 44 | 0.171 | 0.318 |
| Low-elev. | 2004 | 136 | 42 | 37 | 1993 | 165 | 0.068 | 0.251 |
|  | 2005 | 243 | 132 | 55 | 1993 | 165 | 0.122 | 0.790 |
|  | 2006 | 341 | 149 | 36 | 1993 | 165 | 0.171 | 0.892 |

**(B) Wharfedale analyses**

| **Offspring roost** | **Year** | **Number of samples** | | | **Candidate number** | | **Proportion of candidates sampled** | |
| --- | --- | --- | --- | --- | --- | --- | --- | --- |
|  |  | **Males** | **Females** | **Offspring** | **Males** | **Females** | **Males** | **Females** |
| Mid-elev. | 2004 | 30 | 1 | 2 | 146 | 44 | 0.208 | 0.023 |
|  | 2005 | 73 | 4 | 2 | 146 | 44 | 0.507 | 0.091 |
|  | 2006 | 133 | 14 | 6 | 146 | 44 | 0.924 | 0.318 |
| Low-elev. | 2004 | 30 | 42 | 37 | 146 | 165 | 0.208 | 0.251 |
|  | 2005 | 73 | 132 | 55 | 146 | 165 | 0.507 | 0.790 |
|  | 2006 | 133 | 149 | 36 | 146 | 165 | 0.924 | 0.892 |

**Relatedness**

The high level of female natal philopatry observed at the maternity colonies (see Results) suggests there will be relatives of the offspring amongst the candidate mothers. The inclusion of full-siblings to the offspring (*R* = 0.5) among the candidate parents can cause an overestimation of confidence in assignment (Marshall *et al*. 1998). Likelihood tests were carried out to determine if kinship classes could be discriminated and if so, what proportion of full-sibling relationships between adult females and juveniles occurred within roosts. These were carried out using Kinship version 1.2 (Goodnight and Queller, 1999) having compiled allele frequencies and converted files in Kingroup version 2 (Konovalov *et al.*, 2004). The estimated proportions of tests resulting in type II errors (falsely accepting the null hypothesis) at P = 0.05 were obtained by simulations run for 10,000 pairs using population allele frequencies. There was substantial overlap in the distribution of simulated *R*-values. Predicted type II errors were high for tests of whether pairs of adult females and offspring were significantly more likely to be mother-offspring/full-sibling (primary hypothesis) or half-sibling (null hypothesis), for all years (range 0.439 to 0.458); they were also high for tests of half-sibling (primary hypothesis) versus unrelated (null hypothesis) (range 0.297 to 0.303). However, they were low for tests of mother-offspring/full-sibling (primary hypothesis) versus unrelated (null hypothesis) (range 0.014 to 0.016). Therefore only tests for whether pairs of adult females and juveniles were significantly more likely to be mother-offspring/full-sibling than unrelated could be used to assess the proportion of full-siblings to offspring amongst the candidate females. This proportion was calculated by averaging the number of significant *R* = 0.5 relationships minus one (to account for the mother-offspring relationship) per offspring, and dividing this by the number of adult females. This did not exceed 1 % for any year, so this was entered into the Cervus simulation as the proportion of candidate females with an *R* = 0.5 relatedness value to the offspring.

**Paternity assignment to male group**

A program written by Burland *et al.* (2001) used a Bayesian approach to assign probabilities of parentage to different groups. Senior *et al.* (2005) modified this program to estimate the mating success of males from four groups in the Yorkshire Dales in relation to offspring born at a single maternity colony. The program was further adapted for this study to estimate the mating success of males from four groups in relation to offspring born in the low-elevation roosts. The main modifications were the redefinition of male groups, incorporating the effect of sampling offspring in three years and adjusting mutation rates.

The male groups were redefined so that:

*Group 1 = upper-elevation*; these were males from the Buckden and Kettlewell roosts

*Group 2 = mid-elevation*; males from the Grassington roost

*Group 3 = low-elevation*; males from either Addingham Low Mill or Ilkley Cemetery

*Group 4 = swarming*; any males that were *not* caught at Wharfedale roosts and could only have contact with Wharfedale females at swarming sites, including males caught at Wensleydale summer sites and all swarming sites. Should a male have been caught at both a Wharfedale roost and a swarming site, it was entered into its relevant roost group. However, the program took into account that males from Groups 1 to 3 may also contribute to Group 4 (see below).

The female group was made up of individuals from the low-elevation roosts (Addingham Low Mill and Ilkley Cemetery). As with the paternity assignment to individuals, it was assumed that mother and offspring shared the same roost. Both female and male groups comprised genotyped and ‘unknown’ individuals (i.e. individuals that were not caught), to represent all bats whether they had been sampled or not. A sample summary is given in Table 4.

**Table 4. Paternity assignment to male group.**

Total number of male, female and offspring samples used from each area, with breakdown by year (2004, 2005, 2006) in italics. Details for prior distributions of population sizes for males and females: mean with standard deviation and minimum population size estimates.

| **Area** | **Class** | **Samples** | | **Mean (sd)** | **Minimum** |
| --- | --- | --- | --- | --- | --- |
| Upper-elev. | Male | 63 | *23, 18, 22* | 89.9 (8.4) | 84.1 |
|  | Female | - | - | - | - |
|  | Offspring | - | - | - | - |
| Mid-elev. | Male | 19 | *2, 10, 7* | 31.0 (18) | 25.5 |
|  | Female | - | - | - | - |
|  | Offspring | - | - | - | - |
| Low-elev. | Male | 16 | *0, 7, 9* | 24.6 (19) | 19.5 |
|  | Female | 149 | *42, 90, 17* | 165 (19) | 159.5 |
|  | Offspring | 128 | *37, 55, 36* |  |  |
| Swarming | Male | 209 | *98, 73, 38* | 1848 (250) | 516.4 |
|  | Female | - | - | - | - |
|  | Offspring | - | - | - | - |

To account for the effect of year (different individuals would be available as putative parents to different years’ offspring), genotyped individuals were only included as possible parents if they were available as adults (i.e. one or more years old). Also, the number of unknown individuals in each roost was decreased each year as the number of genotyped individuals increased per year, so the total number of individuals at each roost was assumed to remain constant from year to year.

The parameters (θ) to be estimated to form the model were *P_i_* and *N_i_*. *P_i_* were the proportions of offspring whose fathers were drawn from a particular group (*P*_1_ = proportion of low-elevation offspring whose fathers were from an upper-elevation roost, *P*_2_ = mid-elevation, *P*_3_ = low-elevation, and *P*_4_ = swarming). Proposals for the values of *P*_1_, *P*_2_ and *P*_3_ were taken which generated a proposal for *P*_4_ because *P*_4_ *=* 1 – (*P*_1_ + *P*_2_ + *P*_3_), i.e. the father must come from one of these four groups. *N_i_* were the number of individuals (sampled and unsampled) in each group (*N*_1_ = number of males at upper-elevation roosts, *N*_2_ = mid-elevation, *N*_3_ = low-elevation, *N*_4_ = swarming, *N*_f_ = low-elevation females). These were drawn from the prior distributions of population size estimates.

Prior distributions of *N_i_* were Gaussian with means and variances derived from population size estimates whose details are given in Table 4. For Wharfedale areas, the mean number of individuals and standard errors for roosts were estimated using the Jolly-Seber method, as described above. For the swarming group, the population size was estimated from the capture rate at swarming sites of males also caught in Wharfedale, as it was for the paternity assignment to individual males. The standard deviation of the prior distribution for swarming males was set to 250 to make this prior relatively uninformative. Minimum population size estimates for each group were the products of the numbers of individuals caught between 2004 and 2006, and the proportions of males (or females) in the area. Prior distributions were truncated at these lower limits.

The probability of a genotyped father coming from a group (*dp*) given the model, was calculated using the proposed values of *P*, the numbers of genotyped individuals (*G*) and the number of unknown individuals (*U*), with *N* as the number of known and unknown (*G+U*). The probability of a genotyped father coming from the swarming group (*dp*_4_) takes into account the proportion of males at swarming sites that may also come from groups 1 to 3. P_4_ is the probability that a male parent comes from the swarming group but not from one of the focal roosts. *dp*_5_ is the probability of an unsampled male being a father.

*dp*_1_ = (*P*_1_ x *G*_1_) / *N*_1_

*dp*_2_ = (*P*_2_ x *G*_2_) / *N*_2_

*dp*_3_ = (*P*_3_ x *G*_3_) / *N*_3_

*dp*_4_ = (*P*_4_ x *G*_4_) / *N*_4_ + ((*P*_1_ x *U*_1_) / *N*_4_) + ((*P*_2_ x *U*_2_) / *N*_4_) + ((*P*_3_ x *U*_3_) / *N*_4_)

*dp*_5_ = ((*P*_1_ x *U*_1_) / *N*_1_) + ((*P*_2_ x *U*_2_) / *N*_2_) + ((*P*_3_ x *U*_3_) / *N*_3_) + ((*P*_4_ x *U*_4_) / *N*_4_)

The product of the probability of the offspring genotypes given their parents, and the probability of the parents given the model, gives the overall likelihood of the data, i.e. the probability of the genotypes given the model (Senior *et al*., 2005). This likelihood was combined with the prior probability of *N* to give the final likelihood on which the Metropolis sampling was based. This allowed the posterior distribution of *P*, the proportion of fathers being drawn from a group, to be estimated.

The probability that an individual male from a group was a father to a low-elevation offspring was calculated as the probability that the father was from a particular group divided by the number of individuals within that group (sampled or not), i.e. *I*_1_ = *P*_1_ / *N*_1_.

All ten microsatellite loci were used in this analysis. The program took account of the sex-linkage of Paur03, on the X-chromosome. Error and mutation rates specific to loci were incorporated: locus C113 had a trinucleotide repeat and was set an error rate of 1 %; D15 and E24 had single base pair allele repeats so the error rates for these loci were set at 4 %; all others were dinucleotide repeat loci and error rates were 2 % (although Senior *et al.* (2005) found variation in these rates to have little impact on the final *P* estimates).

The program was run for 100,000 iterations, including a 10,000 sample burn-in period. Starting values of *N* were *N*_1_ = 88, *N*_2_ = 36, *N*_3_ = 20 and *N*_4_ = 1750, females = 170. Starting points of *P* were 0.1 for *P*_1_, *P*_2_ and *P*_3_, and 0.7 for *P*_4_.

The following additional runs were made to ensure the results were robust:

- starting points of *P*_1_, *P*_2_ and *P*_3_ set to 0.001 or 0.3 (*P_4_* = 0.997 or 0.1, respectively),
- the widths of the prior distributions of *N* were increased by multiplying the standard errors of the mean population sizes by 2, 3 and 4.

A null model was run for comparison with the definitive results. This version had invariant *P/N* (i.e. constant individual male reproductive success, regardless of group), so it was designed with equal proportions of fathers coming from each group (i.e. *P*_1_ = (*N*_1_ / ∑*N*), *P*_2_ = (*N*_2_ / ∑*N*), etc.). All additional runs were carried out with 100,000 iterations (including 10,000 iterations of burn-in).

**REFERENCES**

Brownstein MJ, Carpten JD, Smith JR (1996) Modulation of non-templated nucleotide addition by *Taq* DNA Polymerase: Primer modifications that facilitate genotyping. BioTechniques 20: 1004-1010.

Burland TM, Barratt EM, Nichols RA, Racey PA (2001) Mating patterns, relatedness and the basis of natal philopatry in the brown long-eared bat, *Plecotus auritus*. Molecular Ecology 10: 1309-1321.

Don RH, Cox PT, Wainwright BJ, Baker K, Mattick JS (1991) Touchdown PCR to circumvent spurious priming during gene amplification. Nucleic Acids Research 19: 4008-4008.

Goodnight KF, Queller DC (1999) Computer software for performing likelihood tests of pedigree relationship using genetic markers. Molecular Ecology 8: 1231-1234.

Jeffreys H (1961) The Theory of Probability (3 ed.). Oxford. p. 432

Konovalov DA, Manning C, Henshaw MT (2004) KINGROUP: a program for pedigree relationship reconstruction and kin group assignments using genetic markers. Molecular Ecology Notes 4: 779-782.

Pollock KH, Nichols JD, Brownie C, Hines JE (1990) Statistical Inference for Capture-Recapture Experiments. Wildlife Monographs 107: 97.

Senior P, Butlin RK, Altringham JD (2005) Sex and segregation in temperate bats. Proceedings of the Royal Society of London, Series B 272: 2467-2473.

Whitlock R, Hipperson H, Mannarelli M, Burke T (2008) A high-throughput protocol for extracting high-purity genomic DNA from plants and animals. Molecular Ecology Resources 8: 736-741.
