# Supplementary material for: Sexual Segregation and Flexible Mating Patterns in Temperate Bats
Source: PLoS One. 2013 Jan 24;8(1):e54194. doi: 10.1371/journal.pone.0054194 (PMC3554751; doi:10.1371/journal.pone.0054194)
Supplement: Results S1 — Results for paternity assignment to individual males and male groups. (DOCX) [file pone.0054194.s002.docx]

**Supporting Information Results S1**

**Sexual segregation and flexible mating patterns in temperate bats**

**RA Angell, RK Butlin and JD Altringham**

**Paternity assignment to individual males**

**Of 10 mid-elevation offspring** in the analysis, two could be assigned fathers (one with strict confidence, one relaxed). Both fathers (one as part of a parent pair) were caught in the year of mating at roosts outside the mid-elevation area, suggesting that mating occurred at a swarming site. One had spent at least five years in the upper-elevation, until it was caught in a low-elevation roost in 2007, one of only a few adult males to move between areas. The other was caught at a roost on the River Ure in Wensleydale (Figure 1). Bats from summer sites in Wharfedale and Wensleydale are known to use a common swarming site (Dow Cave) between the two dales.

**For the 128 lowland offspring** overall assignment rates were 41% for mothers, 13% for fathers and 14% for parent pairs (48 mothers and 10 fathers assigned with strict confidence, five mothers and six fathers with relaxed confidence, 12 parent pairs with relaxed confidence). All but five assigned mothers were caught at the same roost as the offspring, reflecting the level of roost switching between the two low-elevation roosts.

Eleven assigned males were caught during the year an offspring’s parents would have mated, three at nearby swarming sites. One of these was a known summer resident of the upper-elevation site. Six had only ever been caught at upper-elevation summer sites and mating probably occurred at swarming sites. Two fathers were from the low-elevation roosts and most probably mated with the mothers there.

Seventeen fathers assigned either individually, or as part of a parent pair, were not caught during the year of mating. Since bats show a high degree of site fidelity, capture in previous or later years provides an indication of the site location during the year in question. Four were from the upper-elevation, eight from mid-elevation roost or foraging sites and three were caught at a low-elevation roost the year the offspring were born there. The summer site of two is unknown as they were only ever caught at swarming sites.

In summary, of 71 gentoyped males which had been caught at an upper-elevation site between 2004 and 2006, 11 were assigned paternity. Of 47 genotyped males caught at mid-elevation sites, eight were assigned paternity. Of 17 genotyped males caught at a low-elevation site, five were assigned paternity. Of 217 genotyped males caught at swarming sites, five were assigned paternity. Note that of the 28 males assigned paternity of low-elevation offspring, one male was caught at both an upper-elevation site and a swarming site. Of the 341 genotyped males, 11 were caught in more than one area.

These results suggest that fathers of the low-elevation offspring are drawn from the whole dale and perhaps beyond, with mating occurring in summer roosts and at swarming sites.

**Paternity assignment to male group**

Table 5 shows the outcomes of the additional runs of the paternity program. The results were robust to starting conditions and independent of the width of the prior distributions. The definitive model was favoured over the null model, with invariant probability of paternity per male, (Bayes factor = 3.1, based on the harmonic means of post burn-in log likelihoods, support for the model is ‘strong’ i.e. the definitive model was approximately 22 times more likely than the null model (Jeffreys 1961)).

**Table 5. Results from the paternity assignment program runs with definitive and adjusted variables (starting point and altered slope of prior distribution), and for the null model (invariant *P*).**

**(A) Mean values of posterior distributions of *P* with 95 % support limits below in parenthesis.**

| **Run:** | **Definitive** | **Adjusted** | **Adjusted** | **Adjusted** | **Adjusted** | **Adjusted** | **Null** |
| --- | --- | --- | --- | --- | --- | --- | --- |
| **Starting point:** | **0.1, 0.7** | **0.001, 0.1** | **0.3, 0.1** | **0.1, 0.7** | **0.1, 0.7** | **0.1, 0.7** | **0.1, 0.7** |
| **Prior distribution:** | **normal** | **normal** | **normal** | **shallow, 2x sd** | **shallow, 3x sd** | **shallow, 4x sd** | **flat** |
| P_1_ | 0.0086 | 0.0088 | 0.0087 | 0.0089 | 0.0089 | 0.0088 | 0.0354 |
|  | (0.0000 - 0.0280) | (0.0000 - 0.0290) | (0.0000 - 0.0270) | (0.0000 - 0.0300) | (0.0000 - 0.0290) | (0.0000 - 0.0290) | (0.0300 - 0.0430) |
| P_2_ | 0.0081 | 0.0081 | 0.0080 | 0.0084 | 0.0079 | 0.0081 | 0.0132 |
|  | (0.0000 - 0.0270) | (0.0000 - 0.0270) | (0.0000 - 0.0270) | (0.0000 - 0.0290) | (0.0000 - 0.0250) | (0.0000 - 0.0270) | (0.0100 - 0.0200) |
| P_3_ | 0.0166 | 0.0162 | 0.0159 | 0.0165 | 0.0162 | 0.0164 | 0.0106 |
|  | (0.0030 - 0.0420) | (0.0030 - 0.0410) | (0.0030 - 0.0380) | (0.0030 - 0.0430) | (0.0030 - 0.0400) | (0.0030 - 0.0410) | (0.0070 - 0.0170) |
| P_4_ | 0.9667 | 0.9669 | 0.9674 | 0.9662 | 0.9670 | 0.9667 | 0.9409 |
|  | (0.9330 - 0.9890) | (0.9340 - 0.9890) | (0.9360 - 0.9890) | (0.9320 - 0.9880) | (0.9360 - 0.9880) | (0.9340 - 0.9890) | (0.9280 - 0.9520) |

**(B) Mean values of posterior distributions of *I* (*P*/*N*) with 95 % support limits below in parenthesis.**

| **Run:** | **Definitive** | **Adjusted** | **Adjusted** | **Adjusted** | **Adjusted** | **Adjusted** | **Null** |
| --- | --- | --- | --- | --- | --- | --- | --- |
| **Starting point:** | **0.1, 0.7** | **0.001, 0.1** | **0.3, 0.1** | **0.1, 0.7** | **0.1, 0.7** | **0.1, 0.7** | **0.1, 0.7** |
| **Prior distribution:** | **normal** | **normal** | **normal** | **shallow, 2x sd** | **shallow, 3x sd** | **shallow, 4x sd** | **flat** |
| P_1_/N_1_ | 0.0001 | 0.0001 | 0.0001 | 0.0001 | 0.0001 | 0.0001 | 0.0004 |
|  | (0.0000 - 0.0003) | (0.0000 - 0.0003) | (0.0000 - 0.0003) | (0.0000 - 0.0003) | (0.0000 - 0.0003) | (0.0000 - 0.0003) | (0.0003 - 0.0005) |
| P_2_/N_2_ | 0.0002 | 0.0002 | 0.0002 | 0.0002 | 0.0002 | 0.0002 | 0.0004 |
|  | (0.0000 - 0.0008) | (0.0000 - 0.0008) | (0.0000 - 0.0008) | (0.0000 - 0.0008) | (0.0000 - 0.0007) | (0.0000 - 0.0008) | (0.0003 - 0.0005) |
| P_3_/N_3_ | 0.0007 | 0.0006 | 0.0006 | 0.0006 | 0.0006 | 0.0006 | 0.0004 |
|  | (0.0001 - 0.0017) | (0.0001 - 0.0017) | (0.0001 - 0.0016) | (0.0001 - 0.0017) | (0.0001 - 0.0016) | (0.0001 - 0.0017) | (0.0003 - 0.0005) |
| P_4_/N_4_ | 0.0004 | 0.0004 | 0.0004 | 0.0004 | 0.0003 | 0.0003 | 0.0004 |
|  | (0.0003 - 0.0005) | (0.0003 - 0.0005) | (0.0004 - 0.0005) | (0.0003 - 0.0004) | (0.0003 - 0.0004) | (0.0003 - 0.0004) | (0.0003 - 0.0005) |

**(C) Mean values of posterior distributions of *N* with 95 % support limits below in parenthesis.**

| **Run:** | **Definitive** | **Adjusted** | **Adjusted** | **Adjusted** | **Adjusted** | **Adjusted** | **Null** |
| --- | --- | --- | --- | --- | --- | --- | --- |
| **Starting point:** | **0.1, 0.7** | **0.001, 0.1** | **0.3, 0.1** | **0.1, 0.7** | **0.1, 0.7** | **0.1, 0.7** | **0.1, 0.7** |
| **Prior distribution:** | **normal** | **normal** | **normal** | **shallow, 2x sd** | **shallow, 3x sd** | **shallow, 4x sd** | **flat** |
| N_1_ | 91 | 91 | 91 | 94 | 97 | 101 | 91 |
|  | (85 - 102) | (85 - 103) | (85 - 101) | (85 - 114) | (85 - 121) | (85 - 135) | (85 - 102) |
| N_2_ | 35 | 35 | 36 | 41 | 46 | 38 | 33 |
|  | (26 - 56) | (26 - 56) | (26 - 61) | (26 - 72) | (26 - 95) | (26 - 68) | (26 - 51) |
| N_3_ | 26 | 27 | 26 | 30 | 30 | 29 | 27 |
|  | (20 - 40) | (20 - 42) | (20 - 42) | (20 - 50) | (20 - 63) | (20 - 56) | (20 - 43) |
| N_4_ | 2438 | 2406 | 2375 | 2767 | 2809 | 2858 | 2439 |
|  | (2020 - 2810) | (2050 - 2760) | (1970 - 2730) | (2370 - 2990) | (2380 - 2990) | (2490 - 2990) | (2040 - 2830) |
| N_f_ | 207 | 207 | 211 | 226 | 227 | 228 | 209 |
|  | (189 - 227) | (188 - 229) | (193 - 233) | (202 - 258) | (199 - 259) | (201 - 262) | (191 - 229) |
