# Supplementary material for: Sexual Segregation and Flexible Mating Patterns in Temperate Bats
Source: PLoS One. 2013 Jan 24;8(1):e54194. doi: 10.1371/journal.pone.0054194 (PMC3554751; doi:10.1371/journal.pone.0054194)
Supplement: Figure S1 — Relationship between bat activity and altitude. The relationship between bat activity and altitude based on 18×1 km walked acoustic transects along riverside footpaths in July 2007. Bat activity declined significantly with increasing altitude (df = 16; R2 = 0.2378; P = 0.04). Full details are given in the main article. (DOCX) [file pone.0054194.s003.docx]

**Figure S1. Relationship between bat activity and altitude**.

The relationship between bat activity and altitude based on 18 x 1 km walked acoustic transects along riverside footpaths in July 2007. Bat activity declined significantly with increasing altitude (df = 16; R^2^ = 0.2378; P = 0.04). Full details are given in the main article.
