# Supplementary material for: Sexual Segregation and Flexible Mating Patterns in Temperate Bats
Source: PLoS One. 2013 Jan 24;8(1):e54194. doi: 10.1371/journal.pone.0054194 (PMC3554751; doi:10.1371/journal.pone.0054194)
Supplement: Table S1 — Summary of ringed and recaptured bats. Numbers (N) of M. daubentonii (A) adult males and (B) adult females that were ringed at named Wharfedale roost sites, with the number (N) and proportion (%) which were recaptured at the same roost, or at a different roost. (DOCX) [file pone.0054194.s004.docx]

**Table S1.** **Summary of ringed and recaptured bats.**

Numbers (*N*) of *M. daubentonii* (A) adult males and (B) adult females that were ringed at named Wharfedale roost sites, with the number (*N*) and proportion (%) which were recaptured at the same roost, or at a different roost.

1. **Adult males**

| **Site** | **Ringed at roost** | **Recaptured at same roost** | **Recaptured at different roost** |
| --- | --- | --- | --- |
|  | ***N*** | ***N* (%)** | ***N* (%)** |
| Buckden | 65 | 36 (55.4) | 6 (9.2) |
| Kettlewell | 99 | 50 (50.5) | 2 (2.0) |
| Grassington | 49 | 22 (44.9) | 0 (0.0) |
| Addingham | 5 | 1 (20.0) | 0 (0.0) |
| Ilkley | 8 | 2 (25.0) | 1 (12.5) |
| **Total** | **226** | **111 (49.1)** | **9 (4.0)** |

1. **Adult females**

| **Site** | **Ringed at roost** | **Recaptured at same roost** | **Recaptured at different roost** |
| --- | --- | --- | --- |
|  | ***N*** | ***N* (%)** | ***N* (%)** |
| Buckden | 1 | 0 (0.0) | 0 (0.0) |
| Kettlewell | 1 | 0 (0.0) | 0 (0.0) |
| Grassington | 70 | 40 (57.1) | 0 (0.0) |
| Addingham | 84 | 32 (38.1) | 2 (2.4) |
| Ilkley | 73 | 46 (63.0) | 2 (2.7) |
| **Total** | **229** | **118 (51.5)** | **4 (1.7)** |
